# Cognitive control & the anterior cingulate cortex: Necessity & coherence

**DOI:** 10.1016/j.neuroimage.2024.120600

**Published:** 2024-04-02

**Authors:** Lisa Cipolotti, Joe Mole, James K. Ruffle, Amy Nelson, Robert Gray, Parashkev Nachev

**Affiliations:** a Department of Neuropsychology, National Hospital for Neurology and Neurosurgery, London, United Kingdom; b Institute of Neurology, University College London, London, United Kingdom; c Lysholm Department of Neuroradiology, National Hospital for Neurology and Neurosurgery, London, United Kingdom

**Keywords:** Cognitive control, Conflict detection, Stroop, Executive functions, Network lesion-deficit mapping, Conceptual analysis, Anterior cingulate cortex

## Abstract

Influential theories of complex behaviour invoke the notion of cognitive control modulated by conflict between counterfactual actions. Medial frontal cortex, notably the anterior cingulate cortex, has been variously posited as critical to such conflict detection, resolution, or monitoring, largely based on correlative data from functional imaging. Examining performance on the most widely used “conflict” task—Stroop—in a large cohort of patients with focal brain injury (*N* = 176), we compare anatomical patterns of lesion-inferred neural substrate dependence to those derived from functional imaging, meta-analytically summarised. Our results show that whereas performance is sensitive to the integrity of left lateral frontal regions implicated by functional imaging, it does not depend on medial frontal cortex, despite sampling adequate to reveal robust medial effects in the context of phonemic fluency. We suggest that medial frontal cortex is not critically invoked by Stroop and proceed to review the conceptual grounds for rejecting the core notion of conflict-driven cognitive control.

## 1 Introduction

Neural accounts of behaviour require specification of its determinative conditions in causal terms. This seems straightforward for simple behaviours, such as a blink to visual menace, but very difficult for the complex kind, such as spontaneous speech. In the former, the set of necessary and sufficient conditions may be compact enough to be specifiable; in the latter, it is so large as to constitute an indefinite, distributed causal field (Mackie, 1974; Nachev et al., 2019). Furthermore, the wide range in the complexity of plausibly determinative conditions across behaviours raises the question of how the size of the relevant causal field is selected in any specific case.

A tempting solution is to posit a fundamental dichotomy between simple, automatic behaviours determined by narrow sets of conditions, and complex, voluntary behaviours determined by wide causal fields, assigning the selection of the conditioning regime to a cognitive “controller”(Botvinick et al., 2001; Cohen et al., 2000; Koechlin et al., 2003; Miller, 2000; Miller & Cohen, 2001; Ridderinkhof et al., 2004). But endowing a discrete neural substrate with powers close to those of a complete human being is covertly Cartesian (Hacker & Bennett, 2003). For one, it compels us to explain how cognitive control is prompted to intervene: we cannot invoke a “super-controller” without creating an infinite regress (Nachev, 2011). A surprisingly enduring theory assigns this prior task to a “detector”—in its most recent form “evaluator”—of “conflict” arising between competing behaviours (Botvinick & Cohen, 2014; Botvinick et al., 2001; Cohen et al., 2000; Kerns et al., 2004; MacDonald et al., 2000). It is proposed, with apparent support from decades of neuroimaging and modelling research, that the extent of cognitive control is directed by the degree of competition, internally “monitored” by a discrete region of the brain—the anterior cingulate cortex (ACC).

This dichotomised perspective invites behavioural tasks that force a choice between two incompatible actions differing in the complexity of their conditional specification (Nachev, Kennard, & Husain, 2008). Foremost amongst them is the Stroop, introduced as a classic ‘inhibitory’ task (Stroop, 1935). The Stroop Colour-Word part of this test requires participants to name the ink colour in which a colour name is printed when the written name is incongruent (e.g., the word ‘yellow’ is printed in red and the participant must name the colour instead of reading the word). Successful performance has been conceptualised as the ability to inhibit pre-potent responses [e.g., (Cohen et al., 1992; Friedman & Miyake, 2004; Logan, 1994)], working memory [e.g., (Kimberg & Farah, 1993)], general goal maintenance [e.g., (Cohen et al., 1992; West & Baylis, 1998)], as well as the hypothesized control framework invoking conflict monitoring. It is argued that cognitive control is required for Stroop [e.g., (Tolomeo et al., 2016)], underpinned by distributed cortical and subcortical areas often grouped together under the multiple demand network [e.g., (Duncan, 2010; Duncan et al., 2020)], though the task, and others like it, is plausibly decomposable into multiple component processes that may vary with the specific behavioural context (Goel & Dolan, 2003; Goel et al., 2000; Stollstorff et al., 2012). Inferences to neural substrates have primarily rested on correlative data from a very large body of neuroimaging studies extensively meta-analysed [e.g., (Derrfuss et al., 2005; Huang et al., 2020)]. In particular, the Blood Oxygen Level Dependent (BOLD) correlates of Stroop, amongst other conflict tasks, prominently involve the ACC, and show task component modulation in agreement with the predictions of cognitive control theory, where the ACC detects the conflict executive regions proportionately resolve in response. Though the extent of involvement comparative to other regions may be small (Okayasu et al., 2023), functional imaging data is widely held to demonstrate that the ACC is critical, at least to the putative conflict detection process.

A hypothesis, however, is not tested by finding data in agreement with it. Many other explanations are consistent with the observed medial frontal activity, and even if there was none, the complexity of the brain leaves more possible models than anyone could conceivably evaluate. Agreement with one hypothesis is of little consequence if many alternative hypotheses within the realm of possibility remain untested or conform comparably with the data. To test a neuroanatomically framed hypothesis, we must determine if the behaviour is invariant to the integrity of the hypothesised neural substrate. This requires disruptive, not merely correlative data, in human beings mostly limited to lesions of pathological origin (Rorden & Karnath, 2004). Since the richest source of lesion data-—ischaemic stroke—rarely involves the medial wall (Bonkhoff et al., 2021), few disruptive tests of the dependence of Stroop, or any other conflict task, have ever been conducted. Furthermore, the presence of strong pathologically driven correlations across anatomical loci—especially the medial wall—precludes the use of mass-univariate inference, and demands multivariate methods inoperable without substantial volumes of data (Mah et al., 2014; Xu et al., 2018). The few extant lesion-deficit studies are mostly small, of limited coverage, and methodologically vulnerable.

It is therefore unsurprising that lesion-deficit studies of Stroop present an inconsistent picture. The first neuropsychological study of Stroop examined 118 patients with focal lesions and reported that performance was impaired following left dorsolateral prefrontal cortex damage (Perret, 1974). Stuss and colleagues also reported poor Stroop performance after left dorsolateral damage, but also superior medial lesions, particularly involving the right supplementary motor area (Stuss et al., 2001). Others have found impairments on the Stroop following right lateral prefrontal cortex damage (Vendrell et al., 1995). Notably, these two studies included a sizeable number of patients with traumatic brain injury, patients with bilateral lesions, and patients with lesions extending well beyond frontal areas, raising the possibility that widely distributed damage may have hindered focal localization. Very small studies employing Voxel-based Lesion Symptom Mapping (VLSM) have reported impairments on the Stroop on 36 patients with grade 2 or 3 right brain tumors (*n* = 12 with diffuse astrocytoma), who underwent resection of the right cingulate cortex and the middle frontal gyrus (Nakajima et al., 2021).

Others have implicated distributed, bilateral networks. Investigating a large sample of patients with small vessel disease (*n =* 442), Camerino and colleagues found Stroop performance, together with other language and executive abilities, to be sensitive to the integrity of bilateral thalamic radiations, caudate, and forceps minor (Camerino et al., 2021). Focal inferences to cortical function were here limited by the anatomically weakly determinate nature of the lesion pathology. A recent network-level lesion mapping analysis identified a bilateral frontoparietal network in a small number of patients with stroke (*n* = 66; Moore et al., 2024). A study of a larger sample of patients (*n* = 229) with uni- and bilateral lesions associated performance with white matter disconnection of the left frontoparietal nodes within the multiple demand network (Jiang et al., 2023). Surgical resection of low grade gliomas in the right parietal lobe, however, produced no significant decline in Stroop performance in a small sample of 22 patients (Hartung et al., 2021).

Several lesion studies, mainly using VLSM, have converged on the left prefrontal cortex as the critical substrate of Stroop, but without consensus on the finer anatomical detail. A study of a substantial cohort of patients with frontal lesions (*n* = 165) highlighted left dorsolateral frontal cortex (Gläscher et al., 2012). Tsuchida & Fellows investigated the performance of patients with frontal lesions on Evaluated amongst several executive tasks, Tsuchida & Fellows found Stroop to be sensitive to left lateral prefrontal cortex damage (Tsuchida & Fellows, 2013). Geddes and colleagues reported an exaggerated interference effect in Stroop performance in three patients with left ventrolateral PFC lesions (Geddes et al., 2014). We have reported that performance on the Stroop correlates with integrity of left lateral superior and middle frontal gyri (Cipolotti et al., 2016). Others have implicated inferior frontal gyrus (Loosli et al., 2019; Puglisi et al., 2019; Schroeter et al., 2020), and middle frontal gyrus (Puglisi et al., 2019).

The anatomical inconsistency extends to the relationship between Stroop performance and the integrity of the ACC. Several small series with variably ACC-selective lesions have reported mixed results [e.g., (Baird et al., 2006; Fellows & Farah, 2005; Swick & Turken, 2002)]. In 15 patients with bilateral anterior cingulotomy, larger lesion volumes in the ACC correlated with poor performance on the Stroop (Tolomeo et al., 2016). A VLSM study of 63 patients found goal-driven language selection, as measured by Stroop, to be sensitive to the ACC and the left frontal pole (Faulkner & Wilshire, 2020). By contrast, Cipolotti et al. (2016) found no significant difference in Stroop performance between 16 patients with ACC lesions and 15 patients without.

In short, the global localization of Stroop dependence—and the specific contribution of the ACC—remain uncertain. Here we therefore sought to provide a strong test of the necessity of the ACC for Stroop performance. Our claim to sufficient power to address this question rests on five characteristics of the test, in combination uniquely brought to bear on the matter. First, we examine a large set of patients with focal brain injury exhibiting sufficient variance in Stroop performance for its critical substrates to have been adequately evaluated. Second, the lesions in our set demonstrably offer sufficient coverage to detect anatomical dependence on the medial wall. Third, we use Bayesian graph-based multivariate lesion-deficit inference explicitly designed to disentangle behavioural from incidental pathological effects (Cipolotti et al., 2023). Fourth, we use an optimal, data-driven, meta-analytic definition of the region of the medial wall implicated by correlative studies of Stroop. Fifth, we conduct an explicit statistical comparison of models that include or exclude the ACC. These characteristics make ours arguably the most rigorous test of ACC involvement in Stroop in the extant literature.

## 2 Results

### 2.1 Demographics and background tests

Our cohort of patients with frontal and posterior lesions, and healthy control participants, was well-matched for age, sex, and years of education (all *p* >.05; see Table 1). There was no significant difference between the frontal and posterior lesioned groups in the proportion of tumour or stroke aetiologies (both *p* >.05). There were equally no significant aetiological differences between left and right frontal or left and right posterior groups in the proportion of tumour or stroke aetiologies (all *p* >.05).

There were no significant differences between any groups in terms of NART, Incomplete Letters, GNT or TROG scores (all *p* >.05; see Table 2). The frontal lesioned group had significantly lower scores than the posterior lesion and healthy control groups on RAPM [*F*(2,184) = 20.11, *p* <.001; post hoc tests: *t*(119) = −3.08, *p* <.01; *t*(139) = −5.92, *p* <.001, respectively], S fluency [*F*(2,119) = 19.63, *p* <.001; post hoc tests: *t*(109) = −4.21, *p* <.001; *t*(109) = −5.76, *p* <.001, respectively] and Stroop tests [*F*(2,150) = 10.80, *p* <.001; post hoc tests: *t*(110) = −2.54, *p* <.05; *t*(108) = −3.97, *p* <.001, respectively].

In keeping with previous findings, a right frontal effect was observed for RAPM performance. Thus, both left and right frontal patients had significantly lower scores than healthy control participants [*F*(4,182) = 11.84, *p* <.001; post hoc tests: *t*(97) = −3.12, *p* <.001; *t*(107) = −6.75, *p* <.001, respectively] but only right frontal patients had significantly lower scores than posterior lesioned patients [*t*(87) = −4.08, *p* <.001]. Moreover, right frontal patients were significantly more impaired than left frontal [post hoc test: *t*(72) = −2.56, *p* <.01].

Also consistent with previous findings, we observed left frontal effects on phonemic fluency, with both left and right frontal patients exhibiting significantly lower scores than posterior ones [*F*(4,147) = 11.72, *p* <.001; post hoc tests: *t*(72) = −4.46, *p* <.001; *t*(77) = −2.92, *p* <.01, respectively] and healthy control participants [post hoc tests: *t*(72) = −5.56, *p* <.001; *t*(77) = −4.35, *p* <.001, respectively]. Importantly, left frontal patients were significantly more impaired than right frontal [post hoc test: *t*(67) = −2.10, *p* <.05].

### 2.2 Stroop performance

Both left and right frontal groups exhibited significantly lower scores than healthy control participants [*F*(4,148) = 8.20, *p* <.001; post hoc tests: *t*(68) = −4.92, *p* <.001; *t*(80) = −2.70, *p* <.01, respectively]. Importantly, only left frontal patients achieved significantly lower scores than those with posterior lesions [post hoc test: *t*(70) = −3.12, *p* <.001]. This group was also significantly more impaired than the right frontal [post hoc test: *t*(66) = −2.25, *p* <.05].

### 2.3 Graph lesion-deficit mapping

**Fig. 1:**
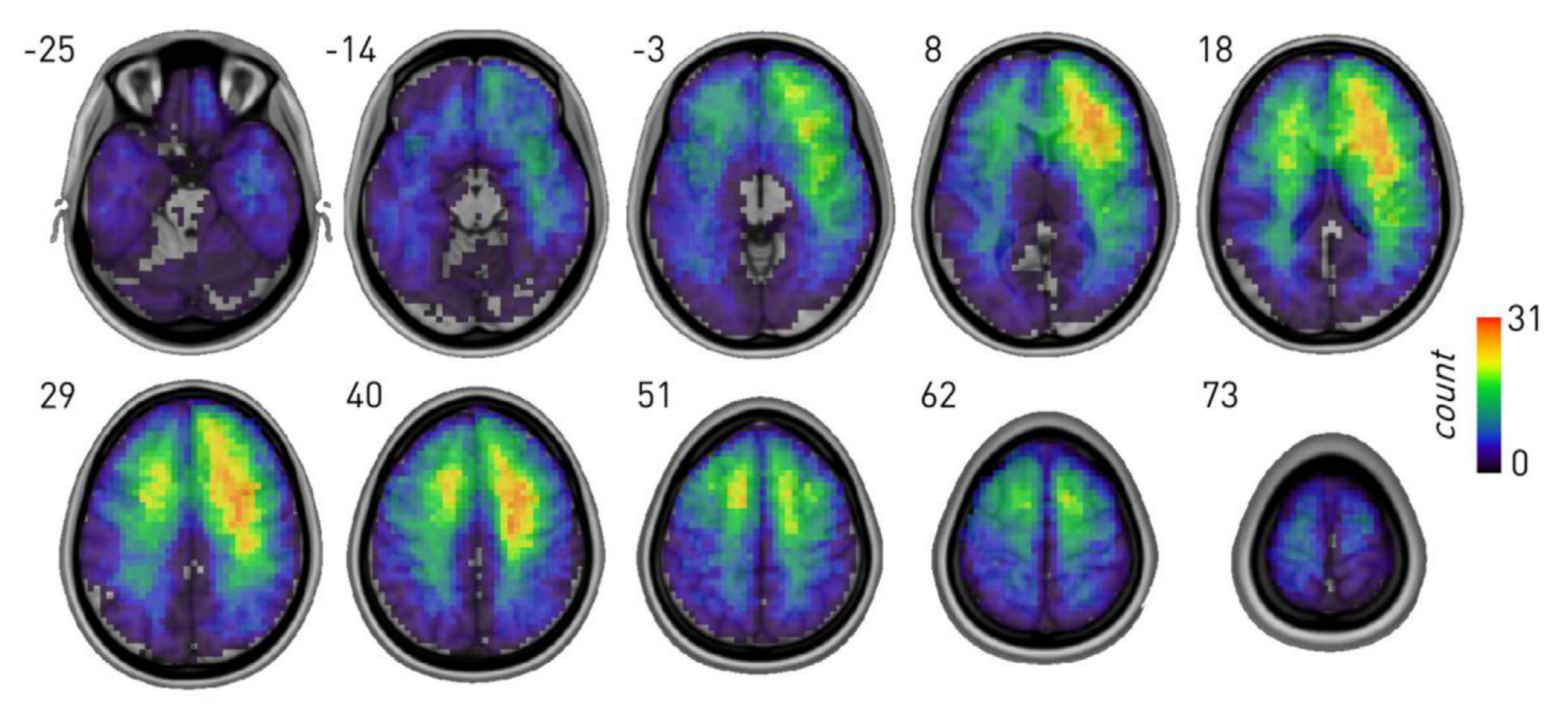
Sum of all lesion images in MNI space. Indices refer to z coordinates in MNI space.

To infer the neural dependents of Stroop, we used graph lesion-deficit mapping based on Bayesian stochastic block modelling (SBM) demonstrably capable of disentangling behavioural from coincidental lesion-pathological effects (Cipolotti et al., 2023) (see Methods). The distribution of lesions in the cohort showed reasonable sampling across the brain, including candidate critical areas (Fig. 1).

**Fig. 2:**
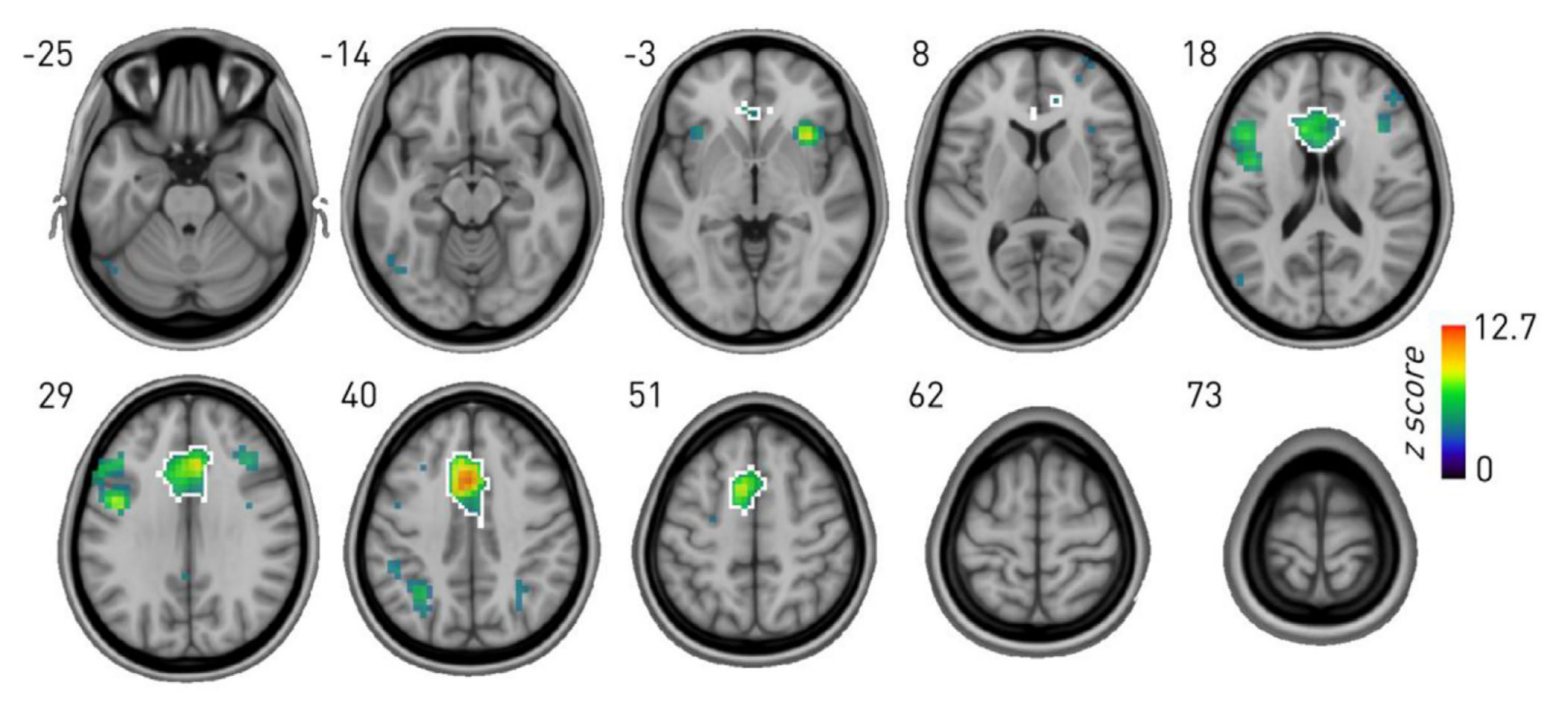
NeuroQuery meta-analytic map of activations associated with the keyphrase “Stroop”. The connected component located on the medial wall, encompassing the ACC, has been outlined in white.

To examine the potential contribution of the ACC, we derived from the NeuroQuery database (Docke’s et al., 2020) a meta-analytic z-score map of consistent activations in extant (overwhelmingly correlative) neuroimaging studies, isolating the connected component located on the medial wall. This coincided closely with the anatomical landmarks of the ACC (Fig. 2, ACC outlined in white).

**Fig. 3:**
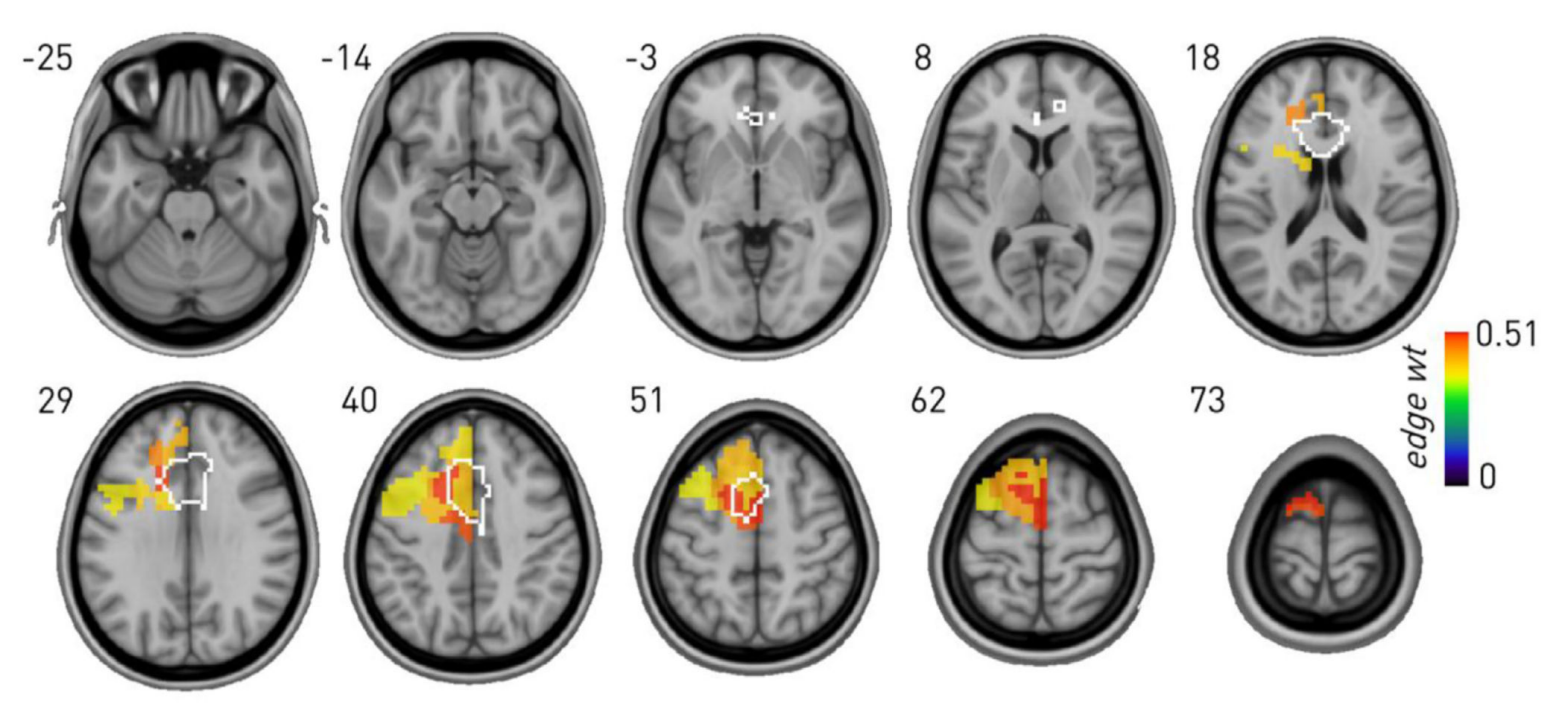
Graph lesion-deficit mapping of phonemic fluency showing the behaviour-associated mean edge weights for SBM-defined regions significantly associated with the behaviour compared with lesion co-occurrence. Note inferred dependence on left prefrontal regions, including within the ACC ROI (outlined in white).

Bayesian SBM models of phonemic fluency performance revealed dependence on medial as well as lateral left frontal areas, including within the ACC region of interest (ROI), demonstrating that the lesion distribution of the test cohort enables mapping of medial frontal areas (Fig. 3). A Bayesian layered SBM of 8719 nodes and 140150 edges achieved sub-stantially lower entropy—432339.440 *vs* 646761.165 nats—-than a null model with weights randomised across the two layers (Fig. 3), providing inferential support for distinguishing fluency from lesional voxel co-occurrence effects. This translates to a posterior odds ratio of the layered formulation being e^214422^ more likely than the non-layered null.

**Fig. 4:**
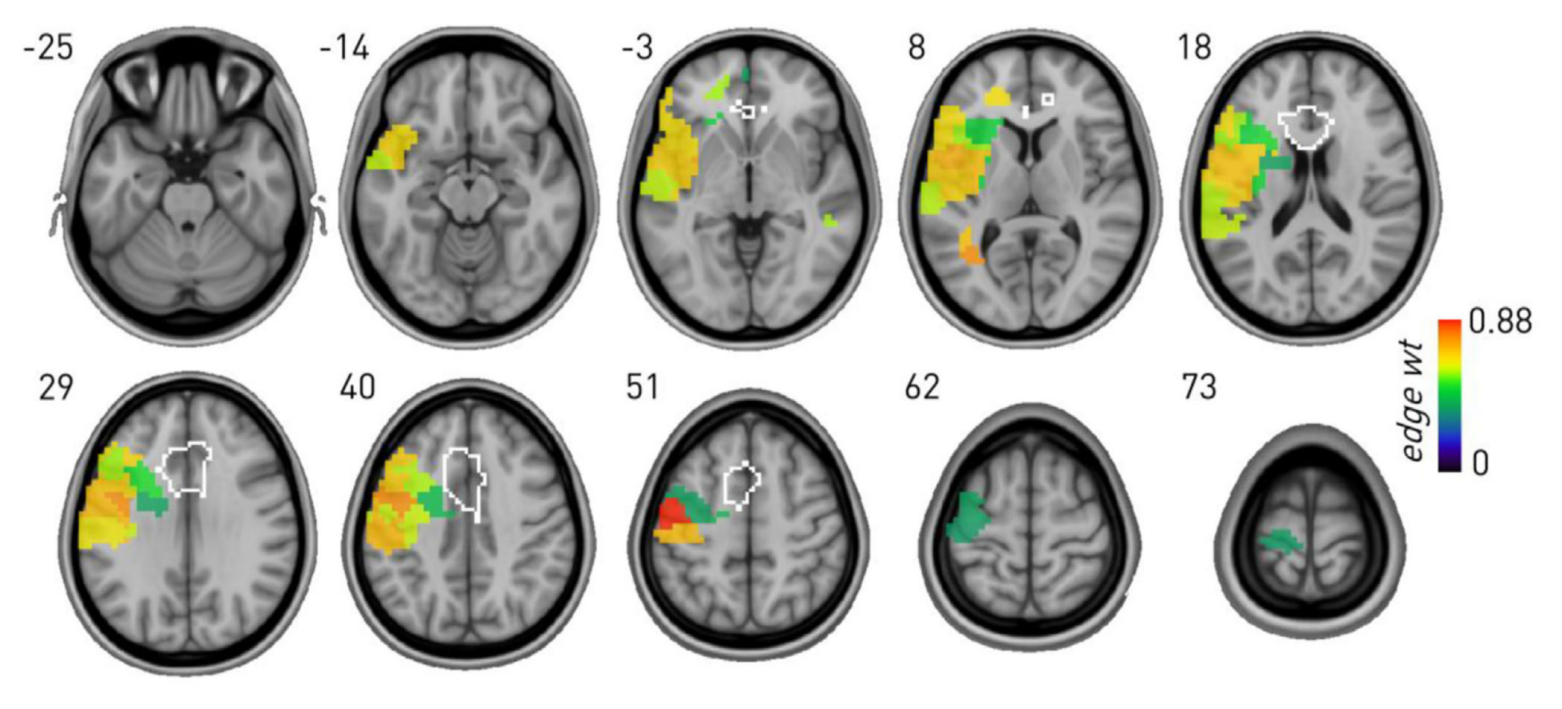
Graph lesion-deficit mapping of Stroop showing the behaviour-associated mean edge weights for SBM-defined regions significantly associated with the behaviour compared with lesion co-occurrence. Note inferred left frontotemporal dependence, excluding the ACC ROI (outlined in white).

By contrast, a SBM of Stroop revealed dependence exclusively on lateral frontotemporal areas, with no evidence of involvement of the ACC ROI (Fig. 4). The layered SBM of 9052 nodes and 142918 edges achieved substantially lower entropy—440889.980 *vs* 671673.462 nats—than a null model with weights randomised across the two layers (Fig. 4), translating to a posterior odds ratio of the layered formulation being e^230783^ more likely than the non-layered null.

### 2.4 Model comparison

To conduct an explicit test of the marginal value of incorporating ACC information in modelling Stroop performance, we conducted Bayesian comparison of predictive models incorporating the SBM-inferred neural dependents, the ACC ROI, or both, using the well-established Widely Applicable Informa-tion Criterion (WAIC) as the measure of goodness-of-fit (smaller = better) (see Methods) (Watanabe & Opper, 2010). Multivariate Bayesian linear regression models (Makalic & Schmidt, 2016) of the lesion data in each compartment, reduced with principal component analysis to five dimensions to minimize collinearity, yielded a WAIC of 6.37 for the SBM inferred Stroop-critical region, and 12.16 for the ACC ROI. Incorporating both sets of localisations yielded a WAIC of 13.27, leaving the Stroop SBM as the best model.

## 3 Discussion

We have shown, in the most rigorous analysis of its kind, that the neural dependents of Stroop do not extend to the regions of the medial wall, including the ACC, commonly implicated by many correlative imaging studies. Our results compel us to conclude that dealing with response conflict—as measured by what is widely considered to be its archetypal test—does not depend on the integrity of the ACC. Here, we examine the grounds for this conclusion and review conceptual criticisms that arguably render the empirical findings inevitable.

The first question is whether Stroop performance is an adequate index of cognitive control. In common with nearly all “conflict” tasks, the critical behavioural contrast here is both riven by multiple confounds such as differential habituation, time-on-task, and conditional complexity, and open to alternative explanations requiring no invocation of cognitive control or anything like it (Fellows & Farah, 2005; Grinband et al., 2011; Nachev, 2011; Nachev et al., 2005; Nachev, Kennard, & Husain, 2008). But if correlative studies of Stroop are directly cited in support of a theory, disruptive studies cannot be discounted by defects inherent in the task itself. Indeed, using lesion-dependent degradation in overall performance as the critical index elides concerns about the spe-cific aspect of cognitive control—its mediation versus adjustment—for which the ACC is supposed to be critical. Either role would predict anatomically specific degradation, and is undermined by its absence.

The second question is whether we have sampled an adequate range of specifically degraded performance. Our lesioned cohort exhibits both wide variation and significantly lower scores than matched healthy control participants. Moreover, the left frontal group showed significantly impaired performance not only when compared to heathy controls but also when directly compared to patients with right frontal or posterior lesions. Though they can never be completely excluded, neither floor nor ceiling effects could plausibly obscure pathological effects here. Equally, we explicitly exclude or model collateral cognitive deficits such as impaired visuospatial function that could independently degrade performance.

The third question is whether performance in our cohort is sensitive to lesion anatomy. Localisation through mass-univariate testing alone provides insufficient grounds to determine this because focal effects may spuriously emerge through lesion patterns driven by the pathological process itself (Mah et al., 2014; Xu et al., 2018). Our graph lesion-deficit approach, however, explicitly disentangles pathologically from behaviourally mediated effects, allowing us to conclude that the former does not adequately explain the observed localisation (Cipolotti et al., 2023). Were no focal effects present, there would be no difference between a layered stochastic block model that distinguishes the spatial distributions of brain lesions from their cognitive effects, and one where the two are randomly allocated. We have robust grounds for inferring localisation, confined to left lateral frontal and prefrontal regions of the brain.

The fourth question is whether our ability to infer regional dependence extends to the medial wall, and adequately accounts for variation across anatomical territories. Lesion deficit mapping is always constrained by the variable coverage—and highly heterogeneous morphology—of the lesions through which the functional anatomical relation is refracted and from which it is inferred. Our method has previously been shown through semi-synthetic validation—the only admissible test—to disentangle behavioural from merely pathological effects across the brain using samples from the same patient population (Cipolotti et al., 2023). Moreover, we have demonstrated in this specific cohort the medial wall dependence of a different task—phonemic fluency—where the anatomy is not in question (Mole et al., under review). We can thus provide direct evidence of the legibility of anatomical relationships involving the medial wall in our specific cohort.

The fifth question is whether we have directly tested the marginal contribution of the medial wall over and above other regions. It is conceivable that Stroop performance depends both on lateral *and* medial substrates, but perhaps differently weighted. Since our graph lesion-deficit model is generative—not discriminatively fitted to the behaviour—it is less susceptible to ignoring weak, collinear signals than most other lesion-deficit methods. Furthermore, our evaluation of the contribution of each candidate region deliberately applies a lenient statistical threshold to minimize Type 2 errors. But here we additionally perform explicit comparison between models of the SBM-inferred left lateral frontal region with and without the addition of medial regions implicated by metaanalytic analysis of correlative studies of Stroop. This formal model comparison reveals no evidence for additional dependence on the integrity of the medial wall. In any event, cognitive control theory posits a central, gating role for the ACC inconsistent with merely subsidiary neural dependence.

In short, our empirical evaluation provides good grounds for concluding that Stroop does not critically involve the ACC. Indeed, since we address the question with data of greater volume and quality and pursue an analysis with finer sensitivity and stricter rigour than previous lesion studies, our conclusion can only be confidently overturned by further, more comprehensive enquiry.

Of course, absence of evidence is not evidence of absence. Failure to find a ghost inside a machine does not guarantee it is not haunted. But the appeal of conducting further empirical studies is tempered by careful consideration of the theoretical underpinnings of cognitive control. Others have identified conceptual vulnerabilities that require reconsideration of its theoretical basis, and at the very least amendment of its fundamental formulation (Goel, 2022). We wish to suggest that the core conceptual framework is open to three lines of criticism arising proximally to its finer technical details: first, that complex behaviour does not necessitate the processes the theory invokes; second, that the notion of detection lacks neural substance; and third, that conflict cannot be defined independently of the entire system, including the putative controller the detector is supposed to inform.

The issue of necessity arises because the full continuity of simple to complex behaviour—and it is a continuity, not a dichotomy—can be instantiated, both theoretically and practically, without invoking the notion of a cognitive controller at all. Large language models (LLMs) can generate text, including in extended dialogue, that spans almost the full range of human behavioural complexity simply by conditioning directly on a wide causal field, and achieve it with comparatively (to a brain) modest computational substrates (Floridi & Chiriatti, 2020). Nothing in the “behaviour” of LLM’s suggests an incapacity to select complex over simple responses, indeed their “executive” abilities—to the substantial extent complex dialogue tests them—are well within the normal range. Equally, nothing in the architecture of LLMs resembles either a discrete mechanism of control or a detector of the need for it, both in their design and the observed artificial neural activation patterns. Large language models are, or course, radically different from real neural substrates, but the critical point is that the observed *behaviour itself* does not necessarily demand a dedicated mechanism for cognitive control, for it is readily instantiable without it.

Second, the notion of “detection” arguably lacks coherence in the specific neural context. One can licitly speak of detection only where the detected and detector are logically dissociable so that *both* its success *and* failure are possible. I can be said to have detected the onset of a flashed visual stimulus because it is possible for it to occur unobserved or unnoticed. The brain is a densely interconnected set of nodes where signals propagate through the network with imperfect fidelity—inevitably subject to noise—but equally so across the entire system. Neither individual neurons nor neuronal ensembles observe each other—they are directly connected—and there is no level of organisation at which the form of neural interaction changes from direct connectivity to anything like observation. Nor is this notion invoked in the artificial neural network architectures—as opposed to the tasks they are designed to perform—that most closely approximate human abilities. Crucially, the discontinuity detection implies is faithless to the nature of real-world behavioural complexity: simple reflexes aside, what Stroop and its kin dichotomize is naturally continuous, lacking logical—not merely empirical—grounds for any binary threshold. Talk of detection here betrays a fundamentally Cartesian notion of an “internal spectator”, with the ACC in place of Descartes’ idea of the soul.

Third, the ground truth for the presence or absence of conflict between a set of actions requires reference not just to the detector but the controller itself, amongst other substrates. The applicable notion of conflict is not merely mechanical—whether two or more actions can be performed simultaneously—or low-level neural—whether their substrates are mutually inhibitory—but *teleological—*how two or more actions relate to the desired goal. For example, mistyping “l” for the last character of the word “kilo” is not a lesser error than mistyping “t” simply because that key is closer to the correct one on the keyboard, in both Euclidean and plau-sible neural “motor” space. A conflict signal capable of usefully informing the controller must reflect the *entire teleologically defined response surface*, for there is no available ground truth for conflict here *but* the full space of actionable possibility and the organism’s goals within it. Determining the parameters of that surface is precisely the task the controller is invoked to solve. The recipient of the conflict signal is thus required to generate it, creating a logical circularity.

**Fig. 5:**
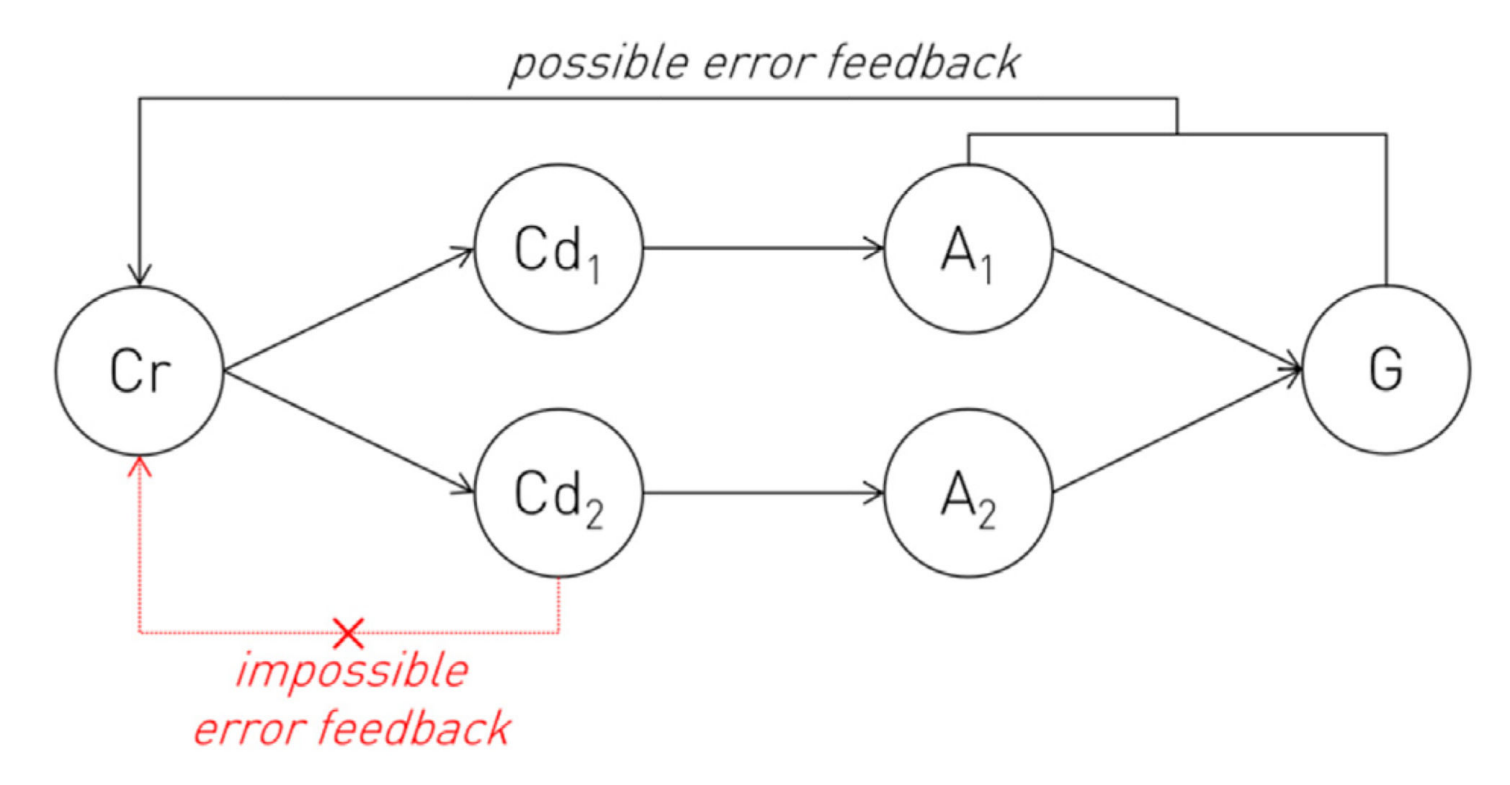
Schematic illustrating the impossibility of “internal” error feedback at a posited controller stage where the behaviour requires learning from success or failure of the achieved goal. A controller, Cr, directs two controlled substrates, Cd, producing two different actions, A, in pursuit of a goal, G. Since it is definitional of a controller that is has knowledge of what it controls, learnt operation of this circuit must receive an error signal at the controlled substrate stage. But this is impossible, for the definition of success or failure in the taskd−achieving the goal−is available only from the final action and its sensorially registered consequences, spanning both controller and controlled.

Indeed, this problem arises in any putatively mechanistic model that posits an “epistemic gap” between controller and controlled component substrates. Consider a simple model where a controller Cr, selects one of two controlled substrates, Cd_1_ or Cd_2_ to produce actions A_1_ or A_2_ in pursuit of some goal G (Fig. 5). It is constitutive of the notion of a controller that it determines the operation of the substrates it controls without involving them in the decision-making, i.e., Cr here selects Cd_1_ or Cd_2_ with all available knowledge of their output. This implies that the controller’s inductive signals—the de-terminants of the decision—are independently generated. But an inductive signal here could arise from only two sources. The first is a non-learnt, pre-specified inductive bias, encoded without any feedback from the system in development or operation. The second is error feedback from the sensorially registered *consequences* of the executed actions with respect to the goal, learnt over time. There is no possible mechanism—information theoretically no possible mechanism—for independent, “internal” error feedback at any intermediate stage, such as the controlled substrate level, for there is no ground truth of what constitutes a correct decision *except at the final output*. The truth of this is reflected in contemporary artificial neural network design, where training is end-to-end unless *external* information is available. Cr here therefore cannot acquire a learnt inductive signal independently of Cd, and so cannot satisfy the definition of a controller over Cd. It is conceivable that there may be general neural properties of Cd—such as the distinct distributional characteristics of Cd_1_ and Cd_2_’s neural activation patterns—that a hypothetical circuit may reinforce by internal feedback, just as the latent representation of a variational autoencoder is driven to conform to a Gaussian distribution (Kingma & Welling, 2013), but such inductive bias still contains no information about the *consequences* of the choice between Cd_1_ and Cd_2_, for all it alters is their distinguishability. Such a controller would still fail to satisfy its criterial definition. It is also conceivable that Cd may be architecturally simple enough to be pre-specified, but only absurdly simple circuits, such as reflexes, have sufficiently few free parameters to be genetically encoded. In any event, such empirical evidence as we have from the persistence of abnormal behaviour following low-level aberrant reinnervation, e.g., in facial nerve damage, is against such a model (Sumner, 1990). In short, a discrete controller-based architecture would have to be non-learnt, pre-specified in a genome that leaves no plausible room for the expressivity necessary to support the full spectrum of goal-directed action.

Note that this hard theoretical constraint does not extend to hierarchical neural models in general, only to those that posit a controller/controlled dichotomy. An end-to-end optimized learning system is free to adopt any organisation that delivers the input/output transformation it seeks to embody, but each component can only be adequately informed—in learnt development—from the only available source of sufficiently expressive error: the output. Every component is here learning from the output, subserviently to it, and the exhibited control is of the system as a whole, not any discrete part.^1^

The primary focus of our paper is an empirical test of the hypothesized neural substrates of cognitive control. Our examination of high-quality lesion data indicates that Stroop performance does not depend on the integrity of the ACC. In any event, the underlying conceptual framework of cognitive control demands re-evaluation of the component processes and the coherence of their posited relations. We hope that our analysis may stimulate future conceptual reflection and empirical enquiry that cast further light on the neural mechanisms underlying complex behaviour.

## 4 Methods

### 4.1 Participants

The patients and healthy controls participated in a study investigating analogical and deductive reasoning (Mole et al., under review). Two-hundred and forty-seven patients with unilateral, focal lesions were prospectively recruited from the inpatient and outpatient stroke and neuro-oncology services at the National Hospital for Neurology and Neurosurgery (NHNN), between the 15th of March 2018 and the 29th of September 2022 (Table 1). Inclusion criteria were: i) presence of a focal stroke or tumour; ii) no history of psychiatric disorders, alcohol or substance abuse, or other neurological disorders; iii) ability to speak and understand English; iv) age between 18 and 80 years; and v) absence of gross perceptual (no neglect, >5th cut-off on the Incomplete Letters test)^33^ or language impairments (>5th %ile on the Graded Naming Test, GNT). Age at assessment, gender, and years of education were recorded. All criteria were determined prior to analysis.

Of these 247 patients, 176 had lesions that fell within frontal (*n* = 102; left frontal 47; right frontal 55) or posterior (*n* = 74; left posterior 32; right posterior 42) areas defined as ≥70% of the total lesion volume, calculated following segmentation of MRI or CT scans obtained during routine clinical care (see ‘Neuroimaging investigations’ section). There was no significant difference between tumour and stroke patients for mean time between injury and neuropsychological assessment (*p* =.30).

Eighty-one healthy control participants, with no neurological or psychiatric history, were recruited to match patients as closely as possible for age, gender, years of education and National Adult Reading Test (NART) scores. The study was approved by The NHNN and Institute of Neurology Joint Research Ethics Committee and conducted in accordance with the ‘Declaration of Helsinki’.

No meaningful sample size calculation is possible in lesion deficit analysis owing to the fundamental nature of the inferential task: enrolment was limited by feasibility.

### 4.2 Behavioural investigations

Participants were assessed with tests administered and scored in the published standard manner.

#### 4.2.1 Background tests

Premorbid optimal level of functioning was assessed using the NART, perception using Incomplete Letters and naming using the GNT. Receptive language was measured using the last 12 item from the Test of Reception of Grammar (TROG) (Bishop, 2003). Fluid intelligence was assessed using RAPM (Raven, 1976). The phonemic fluency test (total number of ‘S’ words generated, excluding errors) was used to assess verbal generation (Spreen & Strauss, 1998).

#### 4.2.2 Stroop test

We used a standard version of the Stroop test (Trenerry, M., Crosson, B., DeBoe, J., & Leber, 1989), which consisted of 112 colour words (red, green, blue or tan), each printed in one of the three incongruent ink colours (i.e., no word is printed in its matching colour). The words were arranged in four equal columns on one A4 sheet. We recorded the total number of ink colours correctly named in 2 min. If participants correctly named all ink colours in less than 2 min, their score was prorated to reflect the number of colours they would have achieved in 2 min. This prorated score was used as a dependent variable for patients and HC. On the basis of normative data, participants’ Stroop performance was classified as impaired if scores were below the 5th percentile (Trenerry, M., Crosson, B., DeBoe, J., & Leber, 1989).

### 4.3 Neuroimaging investigations

Imaging data were available for 237 patients (*n* = 232 MRI, *n* = 5 CT; *n* = 95 frontal, *n* = 71 posterior). MRI scans were obtained on either a 3 T or 1.5 T S scanners in the course of routine clinical care following a diversity of clinically determined protocols outside our control. CT studies were obtained on Toshiba or Siemens spiral scanners. Since the input to the lesion-deficit models is not raw image data but comparatively large, manually-traced, binary lesion masks, we assumed—in common with others in the field—that the effect of variations in acquisition parameters is likely negligible and need not be explicitly modelled. Lesions were traced and independently classified using MIPAV (https://mipav.cit.nih.gov/) by J.M., E.C. and checked by P.N., who was blind to the study results. The lesion masks were non-linearly normalized to Montreal Neurological Institute (MNI) stereotaxic space at 2 × 2 × 2 mm resolution using SPM-12 software with enantiomorphic correction (http://www.fil.ion.ucl.ac.uk) (Nachev, Coulthard, et al., 2008). The lesion distribution is displayed in Fig. 1.

### 4.4 Behavioural analysis

Behavioural analyses were conducted on the 176 patients with lesions that fell within frontal or posterior areas, and healthy controls. Statistical analyses were performed using SPSS version 29. Neuropsychological data were assessed for skewness and kurtosis and tested for normality using the Shapiro—Wilk test.

In the first step of our analysis, we were interested to investigate whether frontal effects were present. In this analysis there was a single factor (group) with three levels (frontal, posterior and healthy control). We used one-way analysis of variance (ANOVA) to compare groups in terms of demographic variables and one-way analysis of covariance (ANCOVA) to compare groups in terms of performance on neuropsychological tests, controlling for age.

Following significant differences, we used post-hoc tests with Bonferroni correction (alpha.05/3 =.016) to compare frontal versus posterior, frontal versus healthy control, and posterior versus healthy control groups. The only exceptions to this were that the healthy control group was not included in the analysis of aetiology, chronicity and lesion volume and Fisher’s exact test was used to compare groups in terms of gender and aetiology.

In the second step of our analysis, we were interested to investigate whether there was evidence of lateralised frontal effects. In this analysis there was a single factor (group) with five levels (left frontal, right frontal, left posterior, right posterior and healthy control). As before, we used one-way ANOVA to compare groups in terms of demographic variables and ANCOVA to compare groups in terms of performance on neuropsychological tests, controlling for age.

Following significant results, we used Bonferroni corrected pairwise comparisons (corrected alpha.05/4 =.0125) to compare each patient group against the healthy control group (i.e., left frontal versus healthy control, right frontal versus healthy control, left posterior versus healthy control, right posterior versus healthy control). Pairwise comparisons were then undertaken to compare left frontal with right frontal, left frontal with posterior and right frontal with posterior. As above, the only exceptions to this were that the healthy control group was not included in the analysis of aetiology, chronicity and lesion volume and Fisher’s exact test was used to compare groups in terms of gender and aetiology.

### 4.5 Graph lesion-deficit mapping

Capturing anatomically distributed neural dependence—and disentangling it from incidental pathologically driven patterns of damage—requires a model of the interactions between anatomical loci. Here we employ a principled and previously validated approach based on statistical models of graphs described and validated in detail elsewhere (Cipolotti et al., 2023; Peixoto, 2015).

In brief, the brain is modelled as a network, where each node is an anatomical location and each edge indexes the extent to which its connected nodes share a set of properties. In the context of lesion-deficit mapping, the properties of interest are the presence of damage, the associated cognitive deficit, and nuisance factors that could confound their relations. Non-parametric Bayesian hierarchical weighted stochastic block modelling is then used to infer the hierarchical organisation of subnetworks of voxels exhibiting dependence on the behavioural score. Such subnetworks or graph communities may be shaped by the neural substrate of the behaviour under study, and/or the pathologically driven anatomical patterns of damage. Layered stochastic block models can be used to disentangle the two distinct types of node connectivity by assigning each type to its own layer, and comparing the goodness-of-fit of the resultant layered model to a null model where these two types of node connectivity are randomly distributed.

In essence, the layered stochastic block model allows us to distinguish correlations between damaged voxels attributable to the lesion-generating pathological process from those associated with the neuropsychological performance of interest. For example, a set of voxels falling within the same vascular territory may be commonly co-lesioned in ischaemic stroke irrespective of the resultant deficit, and if this pattern of co-occurrence is not explicitly modelled, a spurious association with performance will arise. Our task is to identify the set of lesioned voxels whose co-occurrence is explained by their impact on performance over and above the pathological process itself, and this is achieved by explicitly modelling these two structuring effects within distinct layers of the model. Success in this task is indexed by a principled method of model comparison based on minimum description length, essentially a formalisation of Occam’s razor: finding the optimal trade-off between model complexity and goodness-of-fit. In the absence of a ground truth—unlike forecasting, there is no definitive measure of fidelity in lesion-deficit inference—we can validate the model’s ability within semi-synthetic simulations, where clear superiority to standard mass-univariate methods has been demonstrated (Cipolotti et al., 2023).

Since the graph of pairwise relations between anatomical loci at high resolution is too large to be computationally tractable, the imaging data was resampled to 4x4x4 mm resolution, before an adjacency matrix was extracted from each lesion. An undirected, weighted graph combining all individual lesion graphs across all patients was then constructed, composed of nodes corresponding to all voxels of the brain, and edges between all co-lesioned voxels. Separately for models of phonemic fluency and Stroop, edges were weighted by two variables: the count of the number of times a voxel and an adjacent neighbour were damaged together—a lesion cooccurrence weight—and the inverse of the patient’s score in each behavioural test.

An undirected, weighted graph of the brain is derived by accumulating all binary adjacency matrices for all patient lesions, *k*, across the cohort as:

$${\text{A}}_{i,j}^{lesion}=\sum_{k=1}^{N}{\text{A}}_{i,j}^{k}$$

The value within the adjacency matrix, ${\text{A}}_{i,j}^{\text{lesion}}$, is the frequency with which lesions co-occur between nodes *i* and *j*.

Similarly, we define the task weight at edge (*i*; *j*) as

$${\text{A}}_{i,j}^{task}=\sum_{k=1}^{N}\frac{1}{t^{k}\times {\text{A}}_{i,j}^{k}}$$

We only sum over the patients for which the denominator is nonzero. t^*k*^ corresponds to the task performance (here either Stroop or phonemic fluency) for patient *k*.

Edges were filtered to limit analysis to the top 50% connected nodes, removing those with fewer than 3 connections, where sampling was too low to support robust inference. No node self-loops were permitted. We rescaled both lesion cooccurrence and neuropsychological test edge weights to the range 0−1, manually reviewing all edge weight histograms to validate our choice of prior distribution in the model.

For each cognitive test, we proceeded to fit a non-parametric Bayesian hierarchical, weighted stochastic block model incorporating layered and attributed properties implemented in graph-tool (https://graph-tool.skewed.de). We began by fitting a null model, with the two kinds of edge weight—neuropsychological score and lesion co-occurren-ce—randomly distributed across two layers. We then fitted a test model with each type of weight assigned to its own specific layer. Neuropsychological weights were modelled as Gaussian; lesion co-occurrence weights as Poisson distributions. Having initialised a fit, we used simulated annealing to further optimise it, with a default inverse temperature of 1−10. To ensure that equilibration was driven by changes in the entropy criterion, we did not specify a finite number of draws but used a wait step of 100 iterations for a record-breaking event. Model entropy was used to determine if the layered model fit was better than the null, indicating that the inferred community structure distinguished the neuropsychological variable and lesion co-occurrence effects. To visualise the inferred communities, we back-projected the incident edge weights onto the brain with, as well as the first agglomerative levels of the community hierarchy. We statistically tested (via t-testing) whether each set of edges and their corresponding weights passing through a given block of the stochastic block model fit were significantly more ‘lesional’ or ‘task’, retaining only the blocks where the 95% confidence interval of ‘task’ and ‘lesion’ edge arrays did not cross (and hence significantly differed), and zeroing the remainder. Multiple comparisons correction was not used owing to strong correlations between variables and the desire to maximise sensitivity for detecting ACC involvement. Having identified a set of blocks more task and/or lesional in nature, we projected the mean edge weight of a given block into brain space for visualization. In keeping with other studies in the field (Cipolotti et al., 2023; Gläscher et al., 2012), we explicitly chose not to model aetiology, having previously shown this nonadditive for model performance.

### 4.6 Meta-analytic imaging of Stroop

To delineate the region of the medial wall whose activity is correlated with Stroop performance, we derived a meta-analytic functional map from natural language processing of published (overwhelmingly correlative) neuroimaging studies. This was accomplished with the NeuroQuery repository of 13 459 neuroimaging research studies, encom-passing 5,144 activation pattern terms (Docke’s et al., 2020). We retrieved the z-score map, in MNI space, corresponding to the indexed term ‘Stroop’ (thresholded at *z* > 3.1), and isolated the connected component falling within the medial wall, designated as the ACC ROI.

### 4.7 Bayesian model comparison

To determine the marginal contribution of the ACC ROI, we compared multivariate Bayesian penalised regression models of the SBM-inferred communities alone, the ACC ROI alone, and both within the same model. Given the marked collinearity of the regional signals, the dimensionality of each cluster was reduced to five by principal component analysis, capturing 92.89% and 92.30% of the variance, respectively. Each model was specified with a g prior and in Gaussian distribution, and estimated with single chain MCMC sampling over 10 000 samples following a burn-in of 10 000 (Makalic & Schmidt, 2016). We used the Widely Applicable Information Criterion (WAIC) to measure goodness of fit (Watanabe & Opper, 2010).

No part of the study procedures or analyses was preregistered prior to the research being conducted. We report how we determined our sample size, all data exclusions, all inclusion/exclusion criteria, whether inclusion/exclusion criteria were established prior to data analysis, all manipulations, and all measures in the study.

## Supplementary Material

Supplementary files

## Figures and Tables

**Table 1:** Patient characteristics.

	HC (*N* = 81)	Frontal (*N* = 102)	Posterior (*N* = 74)	Left Frontal (*N* = 47)	Right Frontal (*N* = 55)	Left Posterior (*N* = 32)	Right Posterior (*N* = 42)
	Mean	Mean	Mean	Mean	Mean	Mean	Mean
Age (years) (SD)	48.38 (15.62)	47.52 (15.05)	45.99 (15.71)	44.83 (15.59)	49.82 (14.31)	48.31 (14.31)	44.21 (14.71)
Gender (male/female)	35/46	56/48	40/34	26/21	30/25	19/13	21/21
Education (years) (SD)	15.26 (2.54)	14.72 (3.84)	15.46 (2.88)	14.78 (3.49)	14.67 (4.16)	15.12 (2.88)	15.70 (2.90)
Aetiology (tumour/stroke/ abscess/AVM)		78/19/2/3	54/16/2/2	38/7/0/2	40/12/2/1	21/9/1/1	33/7/1/1
Chronicity (days) (SD)		337.39 (830.91)	352.81 (646.84)	302.24 (828.81)	364.07 (839.28)	229.41 (390.97)	455.06 (790.98)
Lesion volume (mm^3^) (SD)		57.32 (79.16)	43.36 (37.59)	56.65 (101.94)	57.95 (50.46)	43.57 (40.06)	43.21 (36.26)

HC = Healthy Controls; SD = standard deviation. There are no significant differences.

**Table 2:** Performance on neuropsychological assessment.

	HC	Frontal	Posterior	Left Frontal	Right Frontal	Left Posterior	Right Posterior
	Mean	Mean	Mean	Mean	Mean	Mean	Mean
NART IQ (SD)	106.00 (9.63)	106.72 (10.07)	109.25 (9.41)	105.31 (10.55)	107.93 (9.61)	108.95 (9.54)	109.42 (9.48)
VOSP IL (/20) (SD)	19.61 (.59)	19.37 (.86)	19.40 (.82)	19.51 (.80)	19.25 (.89)	19.29 (.86)	19.48 (.80)
GNT (/30) (SD)	20.12 (5.19)	18.90 (4.35)	20.01 (5.59)	17.52 (3.92)	20.06 (4.41)	19.17 (6.06)	21.13 (4.54)
TROG (/12) (SD)	9.75 (1.60)	9.41 (1.65)	10.00 (1.65)	9.23 (1.50)	9.58 (1.80)	9.88 (1.65)	10.10 (1.67)
RAPM (/12) (SD)	9.04 (2.10)	6.76 ^a^** ^b^*** (2.45)	8.13 (2.27)	7.56 ^b^*** (2.44)	6.14 ^a^*** ^b^*** ^c^** (2.31)	7.95 (2.01)	8.28 (2.51)
S Fluency (no. Words) (SD)	18.43 (5.30)	12.51 ^a^*** ^b^*** (5 22)	16.64 (4.68)	11.13 ^a^*** ^b^*** ^d^* (5 97)	13.70 ^a^*** ^b^*** (4.20)	15.23 (3.75)	17.28 (4.98)
Stroop (no. Correct) (SD)	108.67 (20.09)	87.29 ^a^* ^b^*** (31 12)	102.84 (32.44)	77.43 ^a^*** ^b^*** ^d^* (33 10)	94.20 ^b^** (28.04)	94.00 (35.67)	108.41 (29.57)

Note: groups are compared using ANCOVA, controlling for age. HC = Healthy Controls; SD = standard deviation; GNT = Graded Naming Test; VOSP IL = Visual Object and Space Perception Battery

Incomplete Letters; SS = scaled score; TROG = Test for Reception of Grammar (Bishop, 2003); RAPM = Raven’s Advanced Progressive Matrices. Scores with significant *p* values are in bold and starred.

a Indicates significant difference from posteriors.

b Indicates significant difference from healthy controls.

c Indicates significant difference from left frontals.

d Indicates significant difference from right frontals.

## Data Availability

The conditions of our ethics approval do not permit public archiving of anonymised study data. Readers seeking access to the data should contact LC or PN. Access will be granted to named individuals in accordance with ethical procedures governing the reuse of sensitive data. Specifically, requestors must complete a formal data sharing agreement. The code for replicating the analysis will be made openly available at https://github.com/high-dimensional/stroop. Legal copyright restrictions do not permit us to publicly archive the full set of stimuli used in this experiment. Readers seeking access to the stimuli are advised to contact the copyright holder (Trenerry, M., Crosson, B., DeBoe, J., & Leber, 1989).

## Footnotes

1 There is a striking parallel here with a surprisingly pervasive logical fault in the philosophy of psychology: the belief that named sensations involve the perception of a private, “inner” object. It remains widely held that one’s experience of (say) pain derives from privileged access to a mental object of it proximally to the expression that determines its name. But whereas one can experience an injury without pain, or experience pain without visible injury, it impossible—logically, not empirically—to expe-rience pain without being aware of it. Similarly, whereas one may fail to notice a flash of light, or perceive a (migrainous) flash where there is none, it is impossible to experience seeing a flash while remaining blind to it. As Wittgenstein showed nearly a century ago, private reference to such an inner object can have no criterion of correctness independently of the external expression that describes it publicly: in our terminology, there is no internal error signal from which correct reference can be learnt (Wittgenstein, 1953). The private neural signal of cognitive control theory is logically as incoherent as the private language signal of Cartesian conceptions of the mind.
