# Supplementary material for: Cognitive control & the anterior cingulate cortex: Necessity & coherence
Source: Neuroimage. Author manuscript; Available in PMC 2025 Oct 1. (PMC7618188; doi:10.1016/j.neuroimage.2024.120600)
Supplement: Supplementary files [file EMS208347-supplement-Supplementary_files.zip › 1-s2.0-S1053811924000958-mmc4.pdf]

Computational limits to the legibility of the imaged human brain

Supplementary Material

James K. Ruffle FRCR MSc[1], Robert J Gray PhD[1], Samia Mohinta MSc[1], Guilherme Pombo MSc[1], Chaitanya Kaul PhD[2], Harpreet Hyare FRCR PHD[1], Geraint Rees FRCP PhD[1], and Parashkev Nachev FRCP PhD[1]

[1]Queen Square Institute of Neurology, University College London, London, UK

[2]School of Computing Science, University of Glasgow, Glasgow, UK

Running title:

Computational limits to the legibility of the imaged human brain

Correspondence to:

Dr James K Ruffle

Email: j.ruffle@ucl.ac.uk

Address: Institute of Neurology, UCL, London WC1N 3BG, UK

Correspondence may also be addressed to:

Professor Parashkev Nachev

Email: p.nachev@ucl.ac.uk

Address: Institute of Neurology, UCL, London WC1N 3BG, UK

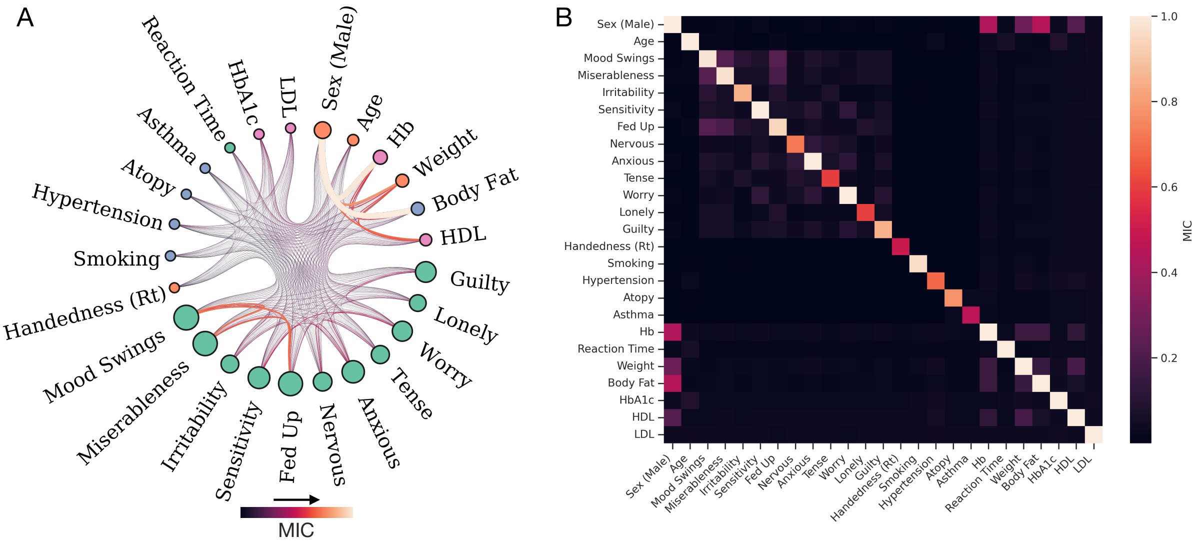

Supplementary Figure 1: Target interrelation. A) Stochastic block model illustrating feature relation by MIC. B) Heatmap of MIC relational values. The process identifies that participant sex holds some degree of informational relationship between their haemoglobin (Hb) concentration, weight, percentage body fat, and high-density lipoprotein (HDL) concentration. Similarly, psychological metrics generally seem to have weak (but nonetheless present) informational relationships to each other, and the same is true between serological variables. Notably the MIC matrix forms the justification for excluding metadata from the same domain as the target for any training run.

Target (Domain)	Biobank Data ID
Sex (C)	https://biobank.ctsu.ox.ac.uk/crystal/coding.cgi?id=9
Age (C)	https://biobank.ctsu.ox.ac.uk/crystal/field.cgi?id=21003
Weight (C)	https://biobank.ctsu.ox.ac.uk/crystal/field.cgi?id=21002
Handedness (C)	http://biobank.ctsu.ox.ac.uk/crystal/field.cgi?id=1707
Mood Swings (P)	https://biobank.ctsu.ox.ac.uk/crystal/field.cgi?id=1920
Miserableness (P)	https://biobank.ctsu.ox.ac.uk/crystal/field.cgi?id=1930
Irritability (P)	https://biobank.ctsu.ox.ac.uk/crystal/field.cgi?id=1940
Sensitivity (P)	https://biobank.ctsu.ox.ac.uk/crystal/field.cgi?id=1950
Fed Up (P)	https://biobank.ctsu.ox.ac.uk/crystal/field.cgi?id=1960
Nervous (P)	https://biobank.ctsu.ox.ac.uk/crystal/field.cgi?id=1970
Anxious (P)	https://biobank.ctsu.ox.ac.uk/crystal/field.cgi?id=1980
Tense (P)	https://biobank.ctsu.ox.ac.uk/crystal/field.cgi?id=1990
Worry (P)	https://biobank.ctsu.ox.ac.uk/crystal/field.cgi?id=2000
Lonely (P)	https://biobank.ctsu.ox.ac.uk/crystal/field.cgi?id=2020
Guilty (P)	https://biobank.ctsu.ox.ac.uk/crystal/field.cgi?id=2030
Reaction Time (ms) (P)	https://biobank.ctsu.ox.ac.uk/crystal/field.cgi?id=20023
Smoking (D)	https://biobank.ctsu.ox.ac.uk/crystal/field.cgi?id=20116
Hypertension (D)	https://biobank.ctsu.ox.ac.uk/crystal/coding.cgi?id=6
Atopy (D)	https://biobank.ctsu.ox.ac.uk/crystal/field.cgi?id=3761
Asthma (D)	https://biobank.ctsu.ox.ac.uk/crystal/field.cgi?id=3786
Body Fat (%) (D)	https://biobank.ctsu.ox.ac.uk/crystal/field.cgi?id=23099
Hb (g/dl) (S)	https://biobank.ctsu.ox.ac.uk/crystal/field.cgi?id=30020
HbA1c (mmol/mol) (S)	http://biobank.ctsu.ox.ac.uk/crystal/field.cgi?id=30750
HDL (mmol/L) (S)	http://biobank.ctsu.ox.ac.uk/crystal/field.cgi?id=30760
LDL (mmol/L) (S)	https://biobank.ctsu.ox.ac.uk/crystal/field.cgi?id=30780

Supplementary Table 1: UK Biobank Data-Fields.

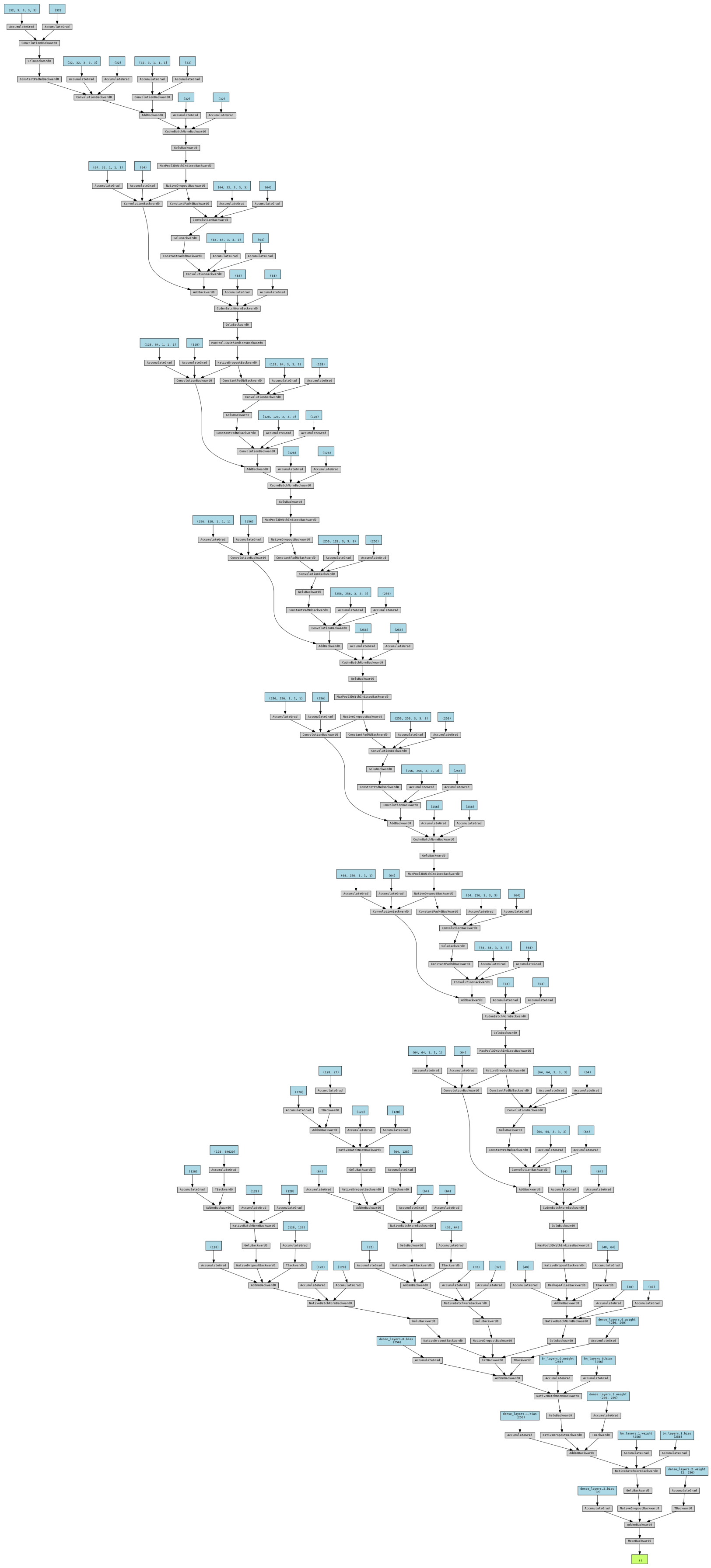

Supplementary Figure 2: Model architectures. Model architectures for the 3D convolutional neural network (CNN), metadata multilayer perceptron (MLP), rsfMRI connectivity MLP, and final prediction MLP.

SEE HTML FILE

Download and open in web browser

Supplementary Figure 3 - Interactive feature mutual information network. Graph of target features, with nodes sized by the mutual information (MI)-weighted eigenvector centrality (EC), and edges sized according to pairwise MI. We depict the top 60% of edges for visualisation purposes. The tab icon in top right of screen allows node labels, edge labels, edge, and node sizes to be modified based upon the variety of parameters fitted. Images can also be exported as static images. Hovering over nodes will show details of the best performing model. Clicking a node will illustrate all results across all models trained for a given target, in order of decreasing performance.

SEE HTML FILE

Download and open in web browser

Supplementary Figure 4 - Interactive feature maximal information coefficient network. Graph of target features, with nodes sized by the maximum information coefficient (MIC)-weighted eigenvector centrality (EC), and edges sized according to the MIC. We depict the top 60% of edges for visualisation purposes. The tab icon in top right of screen allows node labels, edge labels, edge, and node sizes to be modified based upon the variety of parameters fitted. Images can also be exported as static images. Hovering over nodes will show details of the best performing model. Clicking a node will illustrate all results across all models trained for a given target, in order of decreasing performance.

SEE HTML FILE

Download and open in web browser

Supplementary Figure 5 - Interactive feature balanced accuracy network. Graph of target features, with nodes sized by the maximum balanced accuracy across all models (BA), with edges sized according to the mean inverse Euclidean distance of balanced accuracy across all input combinations between each pair of targets. We depict the top 60% of edges for visualisation purposes. The tab icon in top right of screen allows node labels, edge labels, edge, and node sizes to be modified based upon the variety of parameters fitted. Images can also be exported as static images. Hovering over nodes will show details of the best performing model. Clicking a node will illustrate all results across all models trained for a given target, in order of decreasing performance.

SEE XLS FILE

Supplementary dataset – Performance metrics of all 700 models.
